# Evaluating 18s-rRNA LAMP and selective whole genome amplification (sWGA) assay in detecting asymptomatic *Plasmodium falciparum* infections in blood donors

**DOI:** 10.1186/s12936-019-2850-7

**Published:** 2019-06-24

**Authors:** Enoch Aninagyei, Stella Smith-Graham, Alex Boye, Alexander Egyir-Yawson, Desmond Omane Acheampong

**Affiliations:** 1 Department of Biomedical Sciences, School of Allied Health Sciences, University of Cape Coast, Cape Coast, Ghana; 2 School of Biological Sciences, University of Ghana, Legon, Ghana; 3 Department of Medical Laboratory Science, University of Cape Coast, Cape Coast, Ghana; 4 Wellcome Sanger Institute, Hinxton, CB10 1SA UK

**Keywords:** 18s-rRNA-LAMP, Crude DNA LAMP, Purified DNA LAMP, Selective whole genome amplification, *P. falciparum* histidine rich protein 2, *Plasmodium* lactate dehydrogenase, Diagnostic indices, Crude DNA extraction

## Abstract

**Background:**

Undesirable consequences of donor *Plasmodium falciparum* parasitaemia on stored donor blood have been reported. Therefore, it is imperative that all prospective blood donors are screened for *P. falciparum* infections using sensitive techniques. In this study, the sensitivities of microscopy, rapid diagnostic test (RDT), loop-mediated isothermal amplification (LAMP) assay and selective whole genome amplification (sWGA) technique in detecting *P. falciparum* infections in blood donors was assessed.

**Methods:**

Randomly selected blood donors from 5 districts in Greater Accra Region of Ghana were screened for asymptomatic *P. falciparum* infections. Each donor sample was screened with SD Bioline RDT kit for *P. falciparum* histidine rich protein 2 and *Plasmodium* lactate dehydrogenase antigens, sWGA and 18s-rRNA LAMP. Crude DNA LAMP (crDNA-LAMP) was compared to purified DNA LAMP (pDNA-LAMP).

**Results:**

A total of 771 blood donors were screened. The respective overall prevalence of *P. falciparum* in Ghana by microscopy, RDT, crDNA-LAMP, pDNA-LAMP and sWGA was 7.4%, 11.8%, 16.9%, 17.5% and 18.0%. Using sWGA as the reference test, the sensitivities of microscopy, RDT, crDNA-LAMP and pDNA-LAMP were 41.0% (95% CI 32.7–49.7), 65.5% (95% CI 56.9–73.3), 82.6% (95% CI 75.8–88.3) and 95.7% (95% CI 90.1–98.4), respectively. There was near perfect agreement between LAMP and sWGA (sWGA vs. crDNA-LAMP, κ = 0.87; sWGA vs. pDNA-LAMP, κ = 0.96), while crDNA-LAMP and pDNA-LAMP agreed perfectly (κ = 0.91). Goodness of fit test indicated non-significant difference between the performance of LAMP and sWGA (crDNA-LAMP vs. sWGA: *x*^2^ = 0.71, p = 0.399 and pDNA-LAMP vs. sWGA: *x*^2^ = 0.14, p = 0.707). Finally, compared to sWGA, the performance of LAMP did not differ in detecting sub-microscopic parasitaemia (sWGA vs. crDNA-LAMP: *x*^2^ = 1.12, p = 0.290 and sWGA vs. pDNA-LAMP: *x*^2^ = 0.22, p = 0.638).

**Conclusions:**

LAMP assay agreed near perfectly with sWGA with non-significant differences in their ability to detect asymptomatic *P. falciparum* parasitaemia in blood donors. Therefore, it is recommended that LAMP based assays are employed to detect *P. falciparum* infections in blood donors due to its high sensitivity, simplicity, cost-effectiveness and user-friendliness.

## Background

In Ghana, the prevalence of asymptomatic *Plasmodium falciparum* infections is shown to be high among persons over 20-year-old [1] and prospective blood donors [2]. In other parts of Africa, high prevalence of asymptomatic *P. falciparum* parasitaemia in blood donors has also been reported [3–6]. However, blood donors in most malaria-endemic countries in sub-Saharan Africa (SSA) are not tested for malaria [7], despite World Health Organization (WHO) recommendations that all blood donors should be tested for malaria parasites [8].

While some authors have reported that *P. falciparum* can persist for months or years in blood presents a risk to prospective recipients [9, 10], however, others have also argued that the parasite may survive refrigeration for few days [11, 12]. Notwithstanding the ambiguity surrounding the transmissibility of *Plasmodium* parasite through blood transfusion, previous studies have also found storage related untoward haematological and biochemical changes in malaria infected blood donor units [13]. Due to the negative changes impacted to all haematological cell lines and the accumulation of harmful products, it is imperative that all prospective blood donors are screened for malaria parasites to exclude infected donor blood from clinical use. To be able to detect low parasitaemia associated with asymptomatic infections, a sensitive diagnostic technique that is easy to adopt and amenable to blood banks in malaria endemic zones is essential in this regard.

Blood smears are the most utilized method to detect *Plasmodium* parasitaemia. However, this method is limited by its sensitivity and its absolute dependence on the operator. Again, blood smears cannot be performed as a high throughput test due to the huge numbers of donor blood units that are processed by blood banks daily [14]. Due to the aforementioned, there is currently no screening test that is fast, practical, affordable and suitably sensitive for blood banks [15].

LAMP is a multi-primer molecular assay which in comparison to polymerase chain reaction (PCR) is cheaper, simpler and faster, taking out three disadvantages of PCR. LAMP method relies on auto cycling strand-displacement DNA synthesis mediated by *Bst DNA polymerase* enzyme. The principal merit of this method is that LAMP can amplify target DNA without prior denaturation of the DNA template because *Bst DNA polymerase* can be activated at isothermal temperatures to perform DNA template strand displacement and thus the LAMP reactions can be conducted at temperatures 60–65 °C [16].

Selective whole genome amplification (sWGA) technique has been used to increase sub-microscopic DNA levels of *Plasmodium* parasites from infected blood samples [17, 18] and *P. falciparum* genomes from dried blood spots [19]. SWGA preferentially amplifies DNA of pathogens from target and host DNA mixture [20]. SWGA technology is similar to PCR in that a specific portion of the DNA in a sample is enriched. Amplifying an entire genome by SWGA differs technically from amplifying a single gene region by PCR. The two techniques differ by how primers are chosen and the amplification conditions used. The primers used for SWGA bind to DNA sequence motifs that are common in the target genome but not the genomes of other contaminating DNA. The SWGA procedure takes advantage of the inherent differences in the frequencies of sequence motifs among species to designed primers specific to a target species. These primers are then used to selectively amplify the target genomes using phi29 multi-displacement amplification technology [21]. The phi29 polymerase is strand displacing and amplifies DNA from primers with high processivity (up to 70-kbp fragments) and is 100 times less error prone than Taq [22]. Considering the merits of LAMP and sWGA, this study explored these two nucleic acid amplification techniques together with microscopy and RDT in detecting asymptomatic falciparum parasitaemia in blood donors.

## Methods

### Study design

The study involved screening of randomly selected blood donors in selected districts in the Regional capital of Ghana. After obtaining donor consent, blood samples were collected into K_2_-EDTA tube. Each donor sample was screened for *P. falciparum* histidine rich protein 2 (PfHRP2) and *Plasmodium* lactate dehydrogenase (pLDH) proteins. Four dry blood spots were prepared for each sample and the remaining whole blood stored at 4 °C. Each *P. falciparum* positive sample was confirmed by both crude DNA LAMP (crDNA-LAMP) and purified DNA LAMP (pDNA-LAMP) and by selective Whole Genome Amplification (sWGA). Negative RDT and microscopy samples were screened with molecular techniques in pools of 10 samples. Each positive sample pool was resolved by screening each sample individually.

### Blood donor selection, specimen collection and markers screening

Blood donors were recruited in collaboration with the National Blood Service, Ghana, according to national protocols and ethics. Random blood donor samples were collected from five different randomly selected districts in the Greater Accra region, Ghana. Blood donors were sampled from November 2017 to July 2018. All donor specimens included in the study were negative for transfusion-transmissible infections (hepatitis B virus, hepatitis C virus, HIV I&II and *Treponema pallidum*). Five milliliter of donor blood was aseptically collected into K_2_-EDTA tubes. EDTA samples were either processed immediately or stored at 4–6 °C till ready to be processed.

### Specimen processing, screening and transport

K_2_-EDTA donor blood specimens were screened for malaria parasites using SD Bioline PfHRP2/pLDH rapid diagnostic test kit (Gyeonggi-do, Republic of Korea). Thick and thin blood films were made in triplicates and labelled after which dry blood spots (DBS) were prepared for each sample, packaged, labelled and stored at room temperature till they were shipped to Wellcome Sanger Institute, Hinxton, UK for *Plasmodium* parasite detection through genome sequencing using high throughput Hiseq Amplicon Sequencing technique. Morphological identification and parasitaemia were determined for *Plasmodium* parasites using 3% Giemsa staining as described elsewhere [23]. Parasitaemia was estimated by dividing the number of asexual parasites per at least 200 leukocytes and multiplied by the estimated baseline WBC of the patients/μL of blood [24].

### Crude *Plasmodium* parasite DNA (crDNA) extraction

Crude DNA was extracted by the boil and spin method as described by Foundation for Innovation Diagnostics, Geneva, Switzerland [25]. In brief, 60 μL of each well mixed donor whole blood, positive and negative control samples were transferred into separate 1.5 mL Eppendorf tubes pre-filled with 60 μL of extraction buffer (400 mM NaCl, 40 mM Tris pH 6.5, 0.4% SDS). The samples were mixed with the extraction buffer by vortex for 10 s. The tubes were then placed in water bath at 95 °C for 5 min, after which the tubes were centrifuged at 10,000 rpm for 3 min. About 30 μL of the clear supernatant was subsequently transferred to a dilution tube pre-filled with 270 μL of sterile nuclease free water. Crude DNA samples were stored at − 20 °C till ready for amplification.

### Pure DNA (pDNA) extraction using modified Boom’s method

Donor whole blood (250 µL) were dispensed into 2-mL sterile Eppendorf tubes containing 250 µL of lysis buffer (1.6 M GuHCl, 60 mM Tris pH 7.4, 1% Triton X-100, 60 mM EDTA, Tween-20 10%), 50 µL Proteinase-K and 250 µL glass beads). The mixtures were incubated horizontally in a shaker (200 rpm) at 65 °C overnight. To capture DNA, 250 µL of diatomaceous earth solution (1.0 g diatomaceous earth (Sigma Aldrich Chemi GmbH) in 5.0 mL of H_2_O containing 50 µL of 37% (wt/vol) HCl was added to the suspensions and incubated at 37 °C with shaking (200 rpm) for 60 min. The mixtures were centrifuged at 14,000 rpm for 10 s and the resulting pellets were washed twice with 900 µL of 70% ethanol (2–8 °C) followed by 900 µL of acetone. The pellets were dried at 50 °C for 20 min and re-suspended in 100 µL of nuclease free water and centrifuged at 14,000 rpm for 10 s. The purified genomic DNA was stored at 4 °C and later used as templates for malaria LAMP assays. Positive and negative extraction controls were done alongside with the donor blood samples.

### Detection of 18S rRNA by crDNA and pDNA LAMP

The LAMP primers used in this study were designed by Poon et al. [26]. The LAMP assay detects the 18S rRNA target in human *Plasmodium* parasites. In brief, the LAMP reaction volumes were made up of 12.5 μL of reaction mix, 1 μL, 2.6 μL and 1 μL of fluorescent detector, primer mix, *Bst DNA polymerase* respectively (Eiken Chemical, Japan), 2.9 μL of nuclease free water and 5 µL of the crDNA or pDNA extract in 0.2 mL PCR tubes (Fisher Scientific, USA). DNA was amplified, together with 4 extraction controls [2 negative controls (nuclease free water) and 2 positive controls (*Plasmodium* specimen with parasite count of 8000 parasites/µL of blood)], on thermal cycler (Veriti, USA) at constant temperature of 65 °C for 1 h and the *Bst DNA polymerase* inactivated at 80 °C for 5 min. The final reaction results were determined by visual examination of the final greenish yellow colour development. A light brown final colour signified a negative reaction.

### *Plasmodium falciparum* primer design and probe selection

*Plasmodium falciparum* genome primers (probes) were designed to preferentially bind to the *P. falciparum* genome (*P. falciparum* 3D7 genome) using PERL script published by Leichty and Brisson [20] was used to select up to 100 (8–12 mer) primers with a predicted specified melting temperature (≤ 30 °C). Top 50 primers with the highest desired/contaminating (D/C) ratios were selected for further analysis. From these 50, primers with more than three complementary nucleotides at 3′ and 5′ ends were removed to prevent formation of hairpin structures. To prevent primer–primer dimerization, primer pairs with more than three complementary nucleotides at their ends were also removed. A final 28 primers that passed quality control checks were ordered from Integrated DNA Technologies (Coralville, IA) as standard desalting purification with a single modification of phosphorothioate bond between the last two 3′ nucleotides to prevent primer degradation by the Phi29 polymerase exonuclease activity. Individual primers were reconstituted in Tris HCl (pH 8.0) buffer and pooled into three sets (probes) following the D/C ranking described above: the first set consisted of the first 10 primers (Probe_10), the second set consisted of the first 20 primers (Probe_20), and the third set consisted of all 28 primers (Probe_28). The primers used to specifically identify *P. falciparum* in donor blood samples were published by Oyola et al. [27].

### Selective whole genome amplification (sWGA) assay

Selective whole genome amplification (sWGA) was used to detect *Plasmodium* parasites in dry blood samples based on published primers and probes [27]. Briefly, *Plasmodium* DNA in dry blood spot (DBS) samples were extracted using QIAamp DNA Investigator Kit (Qiagen, Germany) following the manufacturer’s protocol. The sWGA reaction was performed in 0.2 mL PCR plates in a reaction volume of 50 μL containing at least 5 ng of template DNA, 1× BSA, 1 mM dNTPs, 2.5 μM of each amplification primer, 1× Phi29 reaction buffer and 30 units of Phi29 polymerase enzyme (New England Biolabs). Amplification was done in “stepdown” protocol consisting of 35 °C for 5 min, 34 °C for 10 min, 33 °C for 15 min, 32 °C for 20 min, 31 °C for 30 min, 30 °C for 16 h then heating at 65 °C for 15 min to inactivate the enzymes prior to cooling to 4 °C. Preceding sequencing, sWGA products were cleaned using Ampure XP beads (Beckman Coulter, UK) and 1.8 volumes of beads per 1 volume of sample were mixed and incubated for 5 min at room temperature. After which the bead/DNA mixture was placed on a magnetic rack to capture the DNA-bound beads. Beads were washed twice with 200 μL of 80% ethanol and the bound DNA eluted with 60 μL of elution buffer. Cleaned amplified DNA products (approx. 0.5–1 μg DNA) were used to prepare DNA library using the NEBNext DNA sample preparation kit (New England Biolabs) for high throughput sequencing. DNA libraries were sequenced using Illumina HiSeq 2500 DNA Sequencer.

### Analysis of sequence data

Identification of *P. falciparum* using sWGA sequence data has been reported by Oyola et al. [27]. *Plasmodium falciparum* sequence reads were analysed and no human DNA reads were involved in the process. In brief, sequence reads were demultiplexed and fastq data files were generated downstream automatically using the onboard PC. Subsequently, reads were first trimmed of low-quality bases from their ends using BioEdit v7.2 after sequence data for each sample was subjected to standard Illumina QC procedures. Approximately, 20 million reads per sample was subjected to detailed analysis for enrichment, quality, content, and coverage. Each dataset was analysed independently by mapping sequence reads to the 3D7 reference genome using Burrows-Wheeler Aligner (BWA) [28]. Subsequently, SAMtools [29] was used to generate coverage statistics from the BWA mapping output. A list of over 1.2 million high-quality single-nucleotide polymorphism (SNP) positions, which were not filtered by gene class or region, but on individual properties of SNPs were used. Genotyping of *P. falciparum* by sWGA was performed using Mpileup to count alleles present in at least five reads (alleles with less than five reads were discarded).

### Data analysis

Percentages were calculated by simple proportions. Prevalence of malaria by each diagnostic technique was done by dividing the number of samples found to be infected by the number of tests done (formula indicated below). The sensitivities, specificities, positive and negative predictive values were determined based on the formulae below and their respective 95% confidence internals as well as the inter-diagnostic technique test of agreement (Cohen’s kappa test of agreement) of each technique were determined by SPSS Version 24 (Chicago, IL, USA).

$${\text{Sensitivity}} = {\text{true positive}}/\left( {{\text{true positive}} + {\text{false negative}}} \right) \times 100\%$$

$${\text{Specificity}} = {\text{true negative}}/\left( {{\text{true negative}} + {\text{false positive}}} \right) \times 100\%$$

$${\text{PPV}} = {\text{true positive}}/\left( {{\text{true positive}} + {\text{false positive}}} \right) \times 100\%$$

$${\text{NPV}} = {\text{true negative}}/\left( {{\text{true negative}} + {\text{false negative}}} \right) \times 100\%$$

where PPV and NPV are positive and negative predictive values respectively.

$${\text{P}}_{{({\text{technique x}})}} = {\text{ number of }}P. \, falciparum{\text{ infected blood donors identified by technique x}}/{\text{total number of donors screened}} \times 100\%$$

where P_(technique x)_ is prevalence of *P. falciparum* parasitaemia determined by either microscopy, rapid diagnostic test, LAMP (crDNA or pDNA) or sWGA.

## Results

### Demographic characteristics and *P. falciparum* parasitaemia in the blood donors

Microscopic, serological and molecular (LAMP and amplicon sequencing) of *Plasmodium* parasitaemia were done in 771 blood donors randomly selected from five districts in the Greater Accra Region of Ghana. The number of blood donors analysed in the Ada East, Ashaiman, Ayawaso, Ga South and Ga West districts were 149 (83.2% males), 164 (82.9% males), 155 (80.6% males), 140 (77.2% males) and 163 (77.9% males), respectively. The overall mean age of the blood donors was 30.7 ± 8.8 years. The respective mean ages of the donors in each district was Ada East (32.4 ± 9.2 years), Ashaiman (32.0 ± 9.5 years), Ayawaso (29.8 ± 9.2 years), Ga South (29.1 ± 8.6 years) and Ga West (30.1 ± 8.9 years). The overall geometric mean of parasites density (minimum–maximum count) was 1652 parasites/µL (505–4798 parasites/µL) and the respective geometric mean of parasites density (minimum–maximum count) for Ada East, Ashaiman, Ayawaso, Ga South and Ga West were 2311 parasites/µL (1578–3151 parasites/µL), 991 parasites/µL (633–1895 parasites/µL), 792 parasites/µL (505–1005 parasites/µL), 2334 parasites/µL (697–4798 parasites/µL) and 1607 parasites/µL (512–4105 parasites/µL). Together with the aforementioned data, the highest educational level and the occupations distribution of the blood donors are presented in Table 1.

**Table 1:** Demographic characteristics and *Plasmodium falciparum* count of blood donors stratified into districts

Demographic variable and parasite count	Ada East District (n = 149)	Ashaiman Municipal (n = 164)	Ayawaso sub-metro (n = 155)	Ga South Municipal (n = 140)	Ga West Municipal (n = 163)
Age mean ± SD (years)	32.4 ± 9.2	32.0 ± 9.5	29.8 ± 9.2	29.1 ± 8.6	30.1 ± 8.9
Gender					
Male n (%)	124 (83.2)	136 (82.9)	125 (80.6)	108 (77.2)	127 (77.9)
Female n (%)	25 (16.8)	28 (17.1)	30 (19.4)	32 (22.8)	36 (22.1)
Highest education					
No formal education	7 (4.7)	7 (4.3)	4 (2.6)	8 (5.7)	1 (0.6)
Junior high school^a^	53 (35.6)	57 (34.7)	46 (29.7)	43 (30.7)	48 (29.4)
Senior high school^b^	49 (32.9)	58 (35.4)	65 (41.9)	57 (40.7)	56 (34.4)
Tertiary	32 (21.5)	34 (20.7)	37 (23.8)	32 (22.8)	58 (35.6)
Other^c^	8 (5.3)	8 (4.9)	3 (1.9)	0 (0.0)	0 (0.0)
Occupation					
Student	24 (16.1)	29 (17.7)	37 (23.8)	32 (23.8)	25 (15.3)
Interns	2 (1.4)	2 (1.2)	2 (1.3)	0 (0.0)	2 (1.2)
Formal workers	20 (13.4)	19 (11.6)	7 (4.5)	12 (8.6)	29 (17.8)
Informal workers^d^	70 (47.0)	79 (48.2)	70 (45.2)	66 (47.1)	62 (38.0)
Trading	26 (17.4)	29 (17.7)	19 (12.3)	19 (13.5)	36 (22.1)
Security officers	7 (4.7)	1 (0.6)	14 (9.0)	9 (6.4)	9 (5.5)
Unemployed	0 (0.0)	5 (3.0)	6 (3.9)	2 (1.4)	0 (0.0)
Parasite density (parasites/µL)					
Geometric mean	2311	991	792	2334	1607
Min–max count	1578–3151	633–1895	505–1005	697–4798	512–4105

^a^Middle school education inclusive

^b^O&A level education inclusive

^c^Others include technical/vocational education

^d^All informal workers except traders

### Microscopic, serological and sub-microscopic prevalence of *P. falciparum* in donor blood

The overall prevalence of *P. falciparum* in blood donors in the Greater Accra region was 7.4% (57/771) by microscopy. The prevalence of *P. falciparum* parasitaemia in donors screened from the districts were: Ada East (10.7%), Ashaiman (6.1%), Ayawaso (3.9%), Ga South (9.3%) and Ga West (7.4%). The combined use of *P. falciparum* histidine-rich protein 2 (PfHRP-2) and *Plasmodium* lactate dehydrogenase (pLDH) rapid diagnostic test increased the overall detection rate by 4.4%. With the exception of Ayawaso sub-metro where there was no increased in the detection rate of microscopy and the RDT, there was 2.4–10.0% increase in the detection rate of the RDT in other districts. Of the 91 RDT positive donor samples, both PfHRP2 and pLDH proteins were detected in 36 (39.6%) of the samples. PfHRP2 antigens but not pLDH were detected in 52 (57.1%) of the samples. Three parasites with HRP2 deletions were suspected. However, genotyping was not done to confirm this suspected deletions.

Loop-mediated isothermal amplification (LAMP) performed on both crude (crDNA-LAMP) and pure (pDNA-LAMP) *Plasmodium* parasite DNA extract detected more infections than microscopy and the RDT. The overall prevalence of *P. falciparum* parasitaemia in blood donors detected by crDNA-LAMP and pDNA-LAMP were 16.9% and 17.5%. Across the five districts, both LAMP assays detected more infections than individual use of microscopy and the RDT. The prevalence of *P*. *falciparum* infections based on crDNA-LAMP and pDNA-LAMP assays in each of the districts were Ada East 24.2% and 25.5%, Ashaiman 14.0% (in both cases), Ayawaso 10.3% (in both cases), Ga South 20.0% and 21.4% and Ga West 16.6% and 17.2%.

Selective whole genome amplification (sWGA) assay performed better than all the other diagnostic methods. With sWGA assay, the overall prevalence of *P*. *falciparum* parasitaemia was 18.0%. The prevalence of *P. falciparum* found in Ada East, Ashaiman, Ayawaso, Ga South and Ga West were 24.8%, 14.0%, 11.0%, 22.1% and 19.0% respectively. All the microscopy detectable specimens were also detected by the RDT and the molecular assays (Table 2). Overall prevalence of malaria sub-microscopic parasitaemia determined by RDT, crDNA-LAMP, pDNA-LAMP and sWGA were 4.4%, 9.5%, 10.1% and 10.6%. The sub-microscopic prevalence by the diagnostic methods in the districts are presented in Table 2.

**Table 2:** Prevalence of *P. falciparum* in blood donors by diagnostic technique

Technique	Overall (n = 771)	Ada East (n = 149)	Ashaiman (n = 164)	Ayawaso (n = 155)	Ga South (n = 140)	Ga West (n = 163)	x^2^ (*p* value)
Microscopy							59.0 (< 0.01)
Positive	57 (7.4%)	16 (10.7%)	10 (6.1%)	6 (3.9%)	13 (9.3%)	12 (7.4%)	
Negative	714 (92.6%)	133 (89.3%)	154 (93.9%)	149 (96.1%)	127 (90.7%)	151 (92.6%)	
Rapid diagnostic test							
Total RDT^+^	91 (11.8%)	24 (16.1%)	14 (8.5%)	6 (3.9%)	27 (19.3%)	20 (12.3%)	20.0 (< 0.01)
PfHRP2^+^/pLDH^+^	36 (39.6%)^a^	9 (37.5%)^a^	7 (50.0%)^a^	2 (33.3%)^a^	10 (37.0%)^a^	8 (40.0%)^a^	
PfHRP2^+^/pLDH^−^	52 (57.1%)^a^	13 (51.2%)^a^	7 (50.0%)^a^	4 (66.7%)^a^	17 (63.0%)^a^	11 (55.0%)^a^	
PfHRP2^−^/pLDH^+^	3 (3.3%)^a^	2 (8.3%)^a^	0 (0.0%)^a^	0 (0.0%)^a^	0 (0.0%)^a^	1 (5.0%)^a^	
Molecular assays							
crDNA-LAMP							0.71 (0.399)
Positive	130 (16.9%)	36 (24.2%)	23 (14.0%)	16 (10.3%)	28 (20.0%)	27 (16.6%)	
Negative	641 (84.1%)	113 (78.5%)	141 (86.0%)	139 (89.7%)	112 (80.0%)	136 (83.4%)	
pDNA-LAMP							0.14 (0.707)
Positive	135 (17.5%)	38 (25.5%)	23 (14.0%)	16 (10.3%)	30 (21.4%)	28 (17.2%)	
Negative	636 (82.5%)	111 (74.5%)	141 (86.0%)	139 (89.7%)	110 (78.6%)	135 (82.8%)	
sWGA							
Positive	139 (18.0%)	37 (24.8%)	23 (14.0%)	17 (11.0%)	31 (22.1%)	31 (19.0%)	
Negative	632 (82.0%)	112 (75.2%)	141 (86.0%)	138 (89.0%)	109 (77.9%)	132 (81.0%)	
Sub-microscopic donor parasitaemia							
Rapid diagnostic test	34 (4.4%)	8 (5.4%)	4 (2.4%)	0 (0.0%)	14 (10.0%)	8 (4.9%)	31.70 (< 0.01)
crDNA-LAMP	73 (9.5%)	20 (13.4%)	13 (7.9%)	10 (6.4%)	15 (10.7%)	15 (9.2%)	1.12 (0.290)
pDNA-LAMP	78 (10.1%)	22 (14.7%)	13 (7.9%)	10 (6.4%)	17 (12.1%)	16 (9.8%)	0.22 (0.638)
sWGA	82 (10.6%)	21 (14.1%)	13 (7.9%)	11 (7.0%)	18 (12.8%)	19 (11.6%)	

*RDT* rapid diagnostic test, *PfHRP2 Plasmodium falciparum* specific histidine rich protein 2, *pLDH* Plasmodium specific lactate dehydrogenase, *PfHRP2*^*+*^*/pLDH*^*+*^ both HRP2 and pLDH detected in donor blood, *PfHRP2*^*+*^*/pLDH*^*−*^ HRP2 but not pLDH detected in donor blood, *PfHRP2*^*−*^*/pLDH*^*+*^ LDH but not HRP2 detected in donor blood (HRP2 gene deletion), *crDNA* crude DNA extract, *pDNA* purified DNA extract, *sWGA* selective whole genome amplification

^a^Percentages calculated from the total number of donors tested positive by the RDT

*x*^2^ is Chi square goodness of fit test statistic. Significance level was determined at p < 0.05

Again, in Table 2, goodness of fit test indicated significant difference between the sensitivities of microscopy (*x*^2^ = 59.0, p < 0.01) and RDT (*x*^2^ = 20.0, p < 0.01) and sWGA. However, the sensitivities of LAMP and sWGA did not differ significantly [(crDNA-LAMP vs. sWGA (*x*^2^ = 0.71, p = 0.399) and pDNA-LAMP vs. sWGA (*x*^2^ = 0.14, p = 0.707)]. Comparing to sWGA, the performance of microscopy (*x*^2^ = 31.70, p < 0.01) differed whilst LAMP did not differ [crDNA-LAMP (*x*^2^ = 1.12, p = 0.290) and pDNA-LAMP (*x*^2^ = 0.22, p = 0.638)] in detecting sub-microscopic parasitaemia.

### Concordance between microscopy and RDT

There was perfect agreement between RDT based on pLDH detection and microscopy. However, microscopy agreed with RDT based on PfHRP2 by a little over 50.0% (Table 3). Overall, microscopy detected 62.6% (57/91) of all RDT positive results.

**Table 3:** Agreement between positive malaria RDT categorization and microscopy results

RDT positive results categorization	Malaria microscopy		
	Concordant	Discordant	% agreement
PfHRP2^+^/pLDH^+^ (n = 36)	36	0	100.00
PfHRP2^+^/pLDH^−^ (n = 52)	18	34	52.9
PfHRP2^−^/pLDH^+^ (n = 3)	3	0	100.0
Total	57	34	

*RDT* rapid diagnostic test, *PfHRP2 Plasmodium falciparum* specific histidine rich protein 2, *pLDH* Plasmodium specific lactate dehydrogenase, *PfHRP2*^*+*^*/pLDH*^*+*^ both HRP2 and pLDH detected in donor blood, *PfHRP2*^*+*^*/pLDH*^*−*^ HRP2 but not pLDH detected in donor blood, *PfHRP2*^*−*^*/pLDH*^*+*^ LDH but not HRP2 detected in donor blood (HRP2 gene deletion)

### Sensitivities, specificities and the predictive values of the diagnostic tests

Using sWGA as reference assay, the respective sensitivities (95% confidence interval) of malaria microscopy, RDT, crDNA-LAMP and pDNA-LAMP was 41.0% (32.7–49.7), 65.5% (65.9–73.3), 82.6% (75.8–88.3) and 95.7% (90.1–98.4). The specificities of microscopy and RDT were 100% but that of crDNA-LAMP and pDNA-LAMP were 99.8 and 99.7% respectively. The positive predictive values (PPV) calculated for microscopy and RDT were 100% whilst their respective negative predictive values (NPV) were 88.5% and 92.9%. The PPV for crDNA-LAMP and pDNA-LAMP were 99.2% and 98.5% respectively while the NPV for crDNA-LAMP and pDNA-LAMP were 95.8% and 99.1% respectively (Table 4).

**Table 4:** Diagnostic indices of the diagnostic techniques using sWGA assay as reference

Technique	TP	TN	FP	FN	Sensitivity (95% CI)	Specificity (95% CI)	PPV (95% CI)	NPV (95% CI)
Microscopy	57	632	0	82	41.0 32.7–49.7	100 99.4–100.0	100 99.4–100.0	88.5 87.0–89.9
Rapid diagnostic test	91	632	0	48	65.5 56.9–73.3	100 99.4–100.0	100 99.4–100.0	92.9 91.3–94.3
crDNA-LAMP	129	614	1	27	82.6 75.8–88.3	99.8 99.1–100.0	99.2 94.8–99.9	95.8 94.2–97.0
pDNA-LAMP	133	630	2	6	95.7 90.1–98.4	99.7 98.8–99.9	98.5 94.3–99.6	99.1 97.9–99.6

*crDNA* crude DNA extract, *pDNA* purified DNA extract, *sWGA* selective whole genome amplification, *TP* true positive, *TN* true negative, *FP* false positive, *FN* false negative, *PPV* positive predictive value, *NPV* negative predictive value

### Inter-diagnostic technique agreement

Cohen’s kappa analysis (Table 5) indicated substantial agreement between microscopy and RDT (κ = 0.74) whilst the agreement between microscopy and LAMP, and microscopy and sWGA were moderate (microscopy and crDNA-LAMP κ = 0.56; microscopy and pDNA-LAMP κ = 0.54) and the agreement between microscopy and sWGA assay was also moderate (κ = 0.53). There was substantial agreement between RDT, and LAMP and sWGA (RDT vs. crDNA-LAMP κ = 0.78; RDT vs. pDNA-LAMP κ = 0.77; RDT vs. sWGA κ = 0.76). While crDNA-LAMP agreed perfectly with pDNA-LAMP (κ = 0.91), crDNA-LAMP almost agreed perfectly with sWGA (κ = 0.87) and pDNA-LAMP agreed perfectly with sWGA (κ = 0.96).

**Table 5:** Cohen’s kappa analysis of the diagnostic methods

Kappa (κ)	Microscopy	RDT	crDNA-LAMP	pDNA-LAMP
Microscopy	–	–	–	–
RDT	0.74	–	–	–
crDNA-LAMP	0.56	0.78	–	–
pDNA-LAMP	0.54	0.77	0.91	–
sWGA	0.53	0.76	0.89	0.96

Modified interpretation to kappa test statistic: 0.01–0.20-slight agreement; 0.21–0.40-fair agreement; 0.41–0.60-moderate agreement; 0.61–0.80-substantial agreement; 0.81–0.90-almost perfect agreement; 0.91–1.00-perfect agreement [21]

## Discussion

*Plasmodium* infections in blood donors are mostly asymptomatic due to competent anti-malaria immunity [30] which improves with age due to previous exposure with different strains of the parasite [31, 32]. Due to the competence of the anti-malarial immunity, *P*. *falciparum* parasite density in asymptomatic infections is mostly low [33]. In this study, microscopy technique missed 59.0% of the overall asymptomatic *P. falciparum* infections indicating a high prevalence of sub-microscopic *P. falciparum* parasitaemia in blood donors. It was observed that microscopy could not detect parasitaemia less than 505 parasites/µL of donor blood. Although microscopy is the goal standard for malaria parasites detection however, this widely used technique has low sensitivity with reported limit of detection between 10 and 100 parasites/μL (approximately 0.0001% parasitaemia) [34–36] and it is also operator dependent [37]. Due to these limitations, it was not surprising that malaria microscopy could not detect close to 60% of *P. falciparum* infection in blood donors. The undetected parasite is likely to persist and provoke storage related cellular and immunological changes which when transfused could be inimical to the recipient.

Due to the aforementioned deficiencies of malaria microscopy, rapid diagnostic test kits (RDTs) were introduce in the 1990s to overcome limitations of microscopy [38]. In agreement with previous studies [34, 39–41], RDT detected 34 more infections than microscopy. However, sensitivity of the RDT was far less than sWGA and LAMP. In asymptomatic infections, the performance of RDTs have been found to be very poor due to very low parasite density [42, 43] and a study have reported decreased sensitivity due to high false-negative results associated with asymptomatic infections [44]. There was concordance between detection of pLDH and microscopy in all the 39 infections. The perfect agreement between pLDH and microscopy makes the detection of pLDH proteins in donor blood a surrogate biomarker for the presence of active and viable *P. falciparum* infections. This is because, *Plasmodium* LDH is known to be glycolytic pathway enzyme secreted by the different Plasmodium species. However, only metabolically active infections have been found to express pLDH [45] because pLDH has been reported in several studies to catalyze the inter-conversion of lactate into pyruvate to produce energy for parasite survivability and levels of pLDH in the blood is found to be directly related to *Plasmodium* density [46–49]. It was not surprising that there was absolute concordance between detection of *P. falciparum* LDH and microscopy. Notwithstanding the foregoing, *P. falciparum* specific LDH is a poor biomarker for detecting *P. falciparum* infections in the sense that, the enzyme has been found to disappear within 24 h of immune activation [50].

The reference selective whole genome amplification (sWGA) detected 139 infections while loop-mediated isothermal amplification assay based on crude DNA (crDNA-LAMP) and loop-mediated isothermal amplification assay based on purified DNA (pDNA-LAMP) detected 130 and 135 infections respectively. Similarly, sWGA, crDNA-LAMP and pDNA-LAMP detected 82, 56 and 78 sub-macroscopic infections, respectively. The agreement between the two LAMP assays and sWGA were almost perfect. Similar to results from previous reports [51, 52], this study found the sensitivity of LAMP assay to be very close to PCR variants. The LAMP assay has been shown to be sensitive enough to detect 1 malaria parasitized red cell [53] and has recently been used in field settings to detect malaria parasites [33, 52, 54] confirming the amenability of this molecular assay at low resourced setting. Although pDNA-LAMP and crDNA-LAMP agreed almost perfectly with the reference assay, the sensitivity of the former was higher than the latter. This could probably be due to high genomic DNA yield by the purified DNA extraction than the boil and spin DNA extraction method. This confirms why previous study suggested the use of high integrity DNA targets for LAMP assay because partial degradation or denaturing of target DNA in the process of specimen boiling during crude DNA extraction is a possibility [55]. Simplifying the DNA extraction protocol to obtain a good DNA yield will definitely make LAMP assay a reliable tool for detecting asymptomatic malaria infections in blood donors. And the perfect agreement, in spite of differences in their diagnostic performance, between crDNA-LAMP and pDNA-LAMP makes crude extraction of *Plasmodium* DNA in asymptomatic infection state reliable DNA source to detect asymptomatic malaria infections in blood donor using LAMP assay.

Moreover, Chi square goodness of fit analysis indicated non-significant differences between the performance of crDNA-LAMP and sWGA as well as between pDNA-LAMP and sWGA. Similarly, the differences between the diagnostic sensitivity of LAMP variants and sWGA in detecting sub-microscopy falciparum parasitaemia was also not significant.

Even though sWGA detected more infections than the LAMP assays, sWGA methodology is comparatively complex requiring equipment sophistication, expensive requiring highly-trained personnel and not amenable to low resource laboratories [19, 20]. In the case of LAMP assay, simple incubators, such as a water baths, block heaters or thermal cyclers, are sufficient for DNA amplification [16] making LAMP assay employable in laboratories with limited resources in areas where malaria is endemic. LAMP assay has also been found to be more rapid, easy-to-perform and cost-effective procedure than PCR-based assays [56]. Again, several studies have reported on the field applicability of LAMP technology [57, 58] due to ease of visual readability of final reactions as a result of accumulation of magnesium pyrophosphate by-products [56]. Finally, LAMP assay has been further simplified by inventing a disc disposable chip that contain all reagents and buffers to perform the assay. The reagent is made of lyophilized amplification reagents and DNA extraction kit [59]. Commercialization of disc disposable LAMP-based chip and its applications in the field of malaria diagnosis will further improve on the merits of LAMP assay.

## Conclusion

PCR based molecular assays remains the most reliable technique for detecting malaria parasites. Because in this study, sWGA detected most infections followed by the two LAMP assays. However, the two LAMP assays agreed perfectly with sWGA and statistically the differences in the performance of LAMP assays and sWGA in detecting asymptomatic malaria infections in blood donors were highly insignificant. However, due to the simplicity, cost-effectiveness, amenability to low resourced laboratory set-up, user-friendliness and applicability of LAMP assays to field settings as well as its rapid turnaround time, LAMP technique should be adopted for routine screening for asymptomatic *P. falciparum* infections prior to blood donation and/or transfusion.

## Data Availability

Study data have been deposited in Harvard Dataverse titled Replication Data for: ‘Detecting asymptomatic *P. falciparum* in blood donors’”, 10.7910/DVN/EACK5V.

## Abbreviations

18s-rRNA: 18 Svedberg ribosomal RNA
crDNA: crude DNA extract
FN: false negative
FP: false positive
LAMP: loop-mediated isothermal amplification
NPV: negative predictive value
pDNA: pure DNA extract
PfHRP2: *P. falciparum* histidine rich protein 2
PfHRP2^−^/pLDH^+^: pLDH but not PfHRP2 detected in donor blood
PfHRP2^+^/pLDH^−^: PfHRP2 but not pLDH detected in donor blood
PfHRP2^+^/pLDH^+^: both PfHRP2 and pLDH detected in donor blood
pLDH: Plasmodium lactate dehydrogenase
PPV: positive predictive value
RDT: rapid diagnostic test
sWGA: selective whole genome amplification
TN: true negative
TP: true positive
κ: kappa
